# Comparison of false-discovery rates of various decoy databases

**DOI:** 10.1186/s12953-021-00179-7

**Published:** 2021-09-18

**Authors:** Sangjeong Lee, Heejin Park, Hyunwoo Kim

**Affiliations:** 1 Department of Computer Science, Hanyang University, Seoul, 06978 Republic of Korea; 2 Center for Supercomputing Applications, Korea Institute of Science and Technology Information, Daejeon, 34141 Republic of Korea

**Keywords:** Target-decoy search, False discovery rate, Reverse decoy database, Shuffle decoy database

## Abstract

**Background:**

The target-decoy strategy effectively estimates the false-discovery rate (FDR) by creating a decoy database with a size identical to that of the target database. Decoy databases are created by various methods, such as, the reverse, pseudo-reverse, shuffle, pseudo-shuffle, and the de Bruijn methods. FDR is sometimes over- or under-estimated depending on which decoy database is used because the ratios of redundant peptides in the target databases are different, that is, the numbers of unique (non-redundancy) peptides in the target and decoy databases differ.

**Results:**

We used two protein databases (the UniProt *Saccharomyces cerevisiae* protein database and the UniProt human protein database) to compare the FDRs of various decoy databases. When the ratio of redundant peptides in the target database is low, the FDR is not overestimated by any decoy construction method. However, if the ratio of redundant peptides in the target database is high, the FDR is overestimated when the (pseudo) shuffle decoy database is used. Additionally, human and *S. cerevisiae* six frame translation databases, which are large databases, also showed outcomes similar to that from the UniProt human protein database.

**Conclusion:**

The FDR must be estimated using the correction factor proposed by Elias and Gygi or that by Kim et al*.* when (pseudo) shuffle decoy databases are used.

**Supplementary Information:**

The online version contains supplementary material available at 10.1186/s12953-021-00179-7.

## Introduction

One of the most important steps in peptide identification is to estimate the false discovery rate (FDR). To estimate the FDR, the target-decoy strategy [1] and the mixture model-based method [2, 3] have been suggested. The target-decoy strategy is easy to implement and effective, so it is frequently used [1].

The target-decoy strategy effectively estimates the FDR by creating a decoy database which is identical in size to the target database. There are various decoy construction methods. The most frequently used is the reverse method, which creates a decoy database by reversing the proteins in the database. The shuffle method, which is often used among stochastic methods, employs a decoy database created by shuffling the amino acids of the target database protein [4]. Recently, the de Bruijn method [5] was proposed, which creates a decoy database using a de Bruijn graph. This method is a repeat-preserving decoy database construction method, which resolves a major problem of the (pseudo) reverse database, namely, that the decoy databases are not random.

In this study, we compared the FDRs of various decoy databases. We created protein-level decoy databases by employing the reverse, shuffle, pseudo-reverse, pseudo-shuffle, and de Bruijn methods. The Comet search engine [6] was used for peptide identification with concatenated target-decoy databases. First, the results were compared for identified peptide-spectrum matches (PSMs) with 1% FDR and without the correction factor. The (pseudo) shuffle method leads to overestimation of the FDR [7, 8] because the ratio of redundant peptides in the target database is significantly different. That is, the numbers of unique (non-redundant) peptides in the target and decoy databases are significantly different. To avoid the overestimation problem of the (pseudo) shuffle method, Elias and Gygi proposed using the correction factor when the FDR is estimated [1, 7]. Therefore, the results were compared for identified PSMs with 1% FDR and with or without the correction factor.

## Methods

### Databases

We used two databases, the UniProt human protein database (version 202,006 comprising 210,556 proteins) and the UniProt *Saccharomyces cerevisiae* protein database (version 202,006 comprising 6,758 proteins), with contaminants added (179 proteins). One reverse decoy database and one pseudo-reverse decoy database were created. In addition, five shuffle decoy, five pseudo-shuffle decoy, and five de Bruijn decoy databases were created in consideration of the variation in these decoy databases. In total, ten decoy databases were created in each protein database. Additionally, we used human and *S. cerevisiae* six frame translation (6FT) databases with UniProt databases and contaminants added. (36,846,527 proteins and 166,062 proteins, respectively).

### Dataset

An MS/MS dataset from 11 human cell lines (A549, GAMG, HEK293, HeLa, HepG2, Jurkat, K562, LnCap, MCF7, RKO, and U2OS, each with three replicates) was obtained using an LTQ-Orbitrap Velos mass spectrometer (Thermo Fisher Scientific, Bremen, Germany) [9]. The HEK293 24-fraction MS/MS dataset was obtained with a Q-Exactive Orbitrap mass spectrometer (Thermo Fisher Scientific, Bremen, Germany) [10]. The *S. cerevisiae* Elite MS/MS dataset was obtained with an Orbitrap Fusion mass spectrometer (Thermo Fisher Scientific, Bremen, Germany) [11]. The *S. cerevisiae* 2DLC MS/MS dataset was obtained using a LTQ-Orbitrap hybrid mass spectrometer (Thermo Fisher Scientific, Bremen, Germany) [12]. Peptide fragmentation was performed using the higher-energy collisional dissociation (HCD) method. Supplementary Table 1 shows the number of spectrum in the human cell lines, the HEK293 24 fraction, in the *S. cerevisiae* Elite and 2DLC datasets.

### Decoy database construction

#### Reverse method

This method is most commonly used when the target-decoy strategy is employed. It creates a decoy database by reversing the proteins of a given target database. For example, if there is a target protein called “AGCKDEFR,” the amino acid present in the protein is reversed to make the decoy protein “RFEDKCGA.”

#### Pseudo-reverse method

This method is identical to the reverse method, but it reverses only the peptides between K and R. For example, if there is a target protein called “AGCKDEFR,” it reverses the amino acids between K and R present in the protein to make the decoy protein “CGAKFEDR.”

#### Shuffle method

This method creates a decoy database by shuffling proteins in the given target database. For example, if there is a target protein called “AGCKDEFR,” the amino acid present in the protein is shuffled to create the decoy protein “KDFERCGA.”

#### Pseudo-shuffle method

This method is identical to the shuffle method but shuffles only the peptides between K and R. For example, if there is a target protein called “AGCKDEFR,” the amino acids between K and R present in the protein are shuffled to make the decoy protein “CAGKEFDR.”

#### De Bruijn method

This method creates a decoy database using a de Bruijn graph from the protein of the given target database. For example, if there is a target protein called “AGCKDEFR,” the graph is implemented with the protein in the k-mer form. The edge, indicating the amino acid, is then randomly changed according to the amino acid probability of the entire database to create the decoy protein “CAGKEDFR.”

### Search engine and parameters

We used Comet as the database search engine. (2019.01 rev. 1 version) [6] The following parameters were used for the human cell line datasets and the HEK293 24-fraction dataset: precursor tolerance = 20 ppm, fragment tolerance = 0.02 Da, NTT (the number of tryptic termini) = 2, maximum missed cleavage = 2, fixed modification = carbamidomethyl on cysteine, variable modification = methionine oxidation, min peptide length = 7, and max peptide length = 45. In addition, the following parameters were used for the *S. cerevisiae* dataset: precursor tolerance = 20 ppm, fragment tolerance = 0.5 Da, NTT = 2, maximum missed cleavage = 2, fixed modification = carbamidomethyl on cysteine, variable modification = methionine oxidation, min peptide length = 7, and max peptide length = 45.

### False discovery rate and correction factor

In general, the target-decoy strategy uses the following equation to estimate the FDR [13]:

$${FDR}_{common}=\frac{\#Decoy}{\#Target}$$

Here, *#Target* denotes the number of target PSMs, and *#Decoy* represents the number of decoy PSMs.

To avoid the overestimation problem, Elias and Gygi proposed using the correction factor when the FDR is estimated. The correction factor can easily be obtained by the methods introduced by Elias and Gygi or by Kim et al. [1, 7, 14]. The method proposed by Elias and Gygi is called *Factor* 1 (RRatio), and it can be carried out using the ratio of the target and decoy PSMs at lower ranks, as follows:

$${Factor}_{1}=\frac{{\#Decoy}_{lower}}{\#{Target}_{lower}}$$

Here, *#Target*_*lower*_ denotes the number of target PSMs at a lower rank, and *#Decoy*_*lower*_ is the number of decoy PSMs at a lower rank. PSMs with a lower rank refer to rank 5 PSMs.

The method proposed by Kim et al. is called *Factor* 2 (UPRatio), and it can be calculated using the ratio of the target to the decoy unique peptides of the database, as follows:

$${Factor}_{2}=\frac{\#UDP}{\#UTP}$$

Here, *#UTP* and *#UDP* are, respectively, the numbers of unique target peptides and unique decoy peptides in the database.

The FDR is estimated with the correction factor as follows:

$${FDR}_{factor}=\frac{\#Decoy}{\#Target}\times \frac{1}{Factor}$$

## Results and discussion

We denote the results with 1% FDR from the reverse, shuffle, pseudo-reverse, pseudo-shuffle, and de Bruijn methods as FDR_R_, FDR_S_, FDR_PR_, FDR_PS_, and FDR_D_, respectively.

### Saccharomyces cerevisiae dataset

**Fig. 1:**
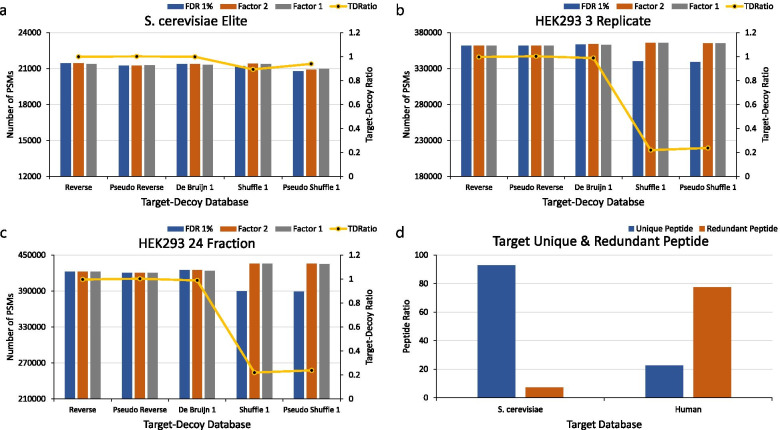
Comparison of the number of PSMs among various databases of typical size and number of target and decoy unique peptides. **a**, **b**, **c** The blue bars show the numbers of PSMs for 1% FDR without the correction factor. The orange bars show the numbers of PSMs for 1% FDR using *Factor* 2. The gray bars show the numbers of PSMs for 1% FDR using *Factor* 1. The yellow line show the ratio of target and decoy unique peptide ratio. **a** The UniProt *S. cerevisiae* protein database and the *S. cerevisiae* Elite dataset. **b** The UniProt human protein database and the HEK293 3-replicate dataset. **c** The UniProt human protein database and the HEK293 24-fraction dataset. **d** The blue bars show the ratios of all peptides to unique peptides. The red bars show the ratios of all peptides to redundant peptides. Comparison of the ratio of unique and redundant peptides for the *S. cerevisiae* and human protein databases

We compared the results for the identified PSMs with the 1% FDR using the *S. cerevisiae* Elite and 2DLC dataset, the protein database, and various decoy databases. As shown in Fig. 1a and in Supplementary Figure 1b, the numbers of PSMs for FDR_R_, FDR_S_, FDR_PR_, FDR_PS_, and FDR_D_ were nearly identical regardless of the decoy construction method. (For consideration of the variation in the shuffle, pseudo-shuffle, and de Bruijn method, the results additional databases, in this case four shuffle, four pseudo-shuffle, and four de Bruijn databases, were compared, as shown in Supplementary Figures 1a and b. There was no variation in the shuffle, pseudo-shuffle, and de Bruijn methods.)

### HEK293 datasets

We used two HEK293 datasets, called the HEK293 3-replicate dataset and the HEK293 24-fraction dataset, and compared the results for the identified PSMs with the 1% FDR among various decoy databases. Figures 1b and 1c show the comparison outcomes for FDR_R_, FDR_S_, FDR_PR_, FDR_PS_, and FDR_D_. The numbers of PSMs for FDR_R_, FDR_PR_, and FDR_D_ were nearly identical, but the numbers of PSMs for FDR_S_ and FDR_PS_ were, in the HEK293 3-replicate dataset, about 6% lower, and in the HEK293 24-fraction dataset, they were about 8% lower than those for FDR_R_, FDR_PR_, and FDR_D_. Hence, the FDR is overestimated for FDR_S_ and FDR_PS_, unlike in the *S. cerevisiae* datasets. Additionally, we used ten cell line datasets and compared the results for the identified PSM with the 1% FDR outcomes among the various decoy databases. Supplementary Figure 2 shows the comparison results for FDR_R_, FDR_S_, FDR_PR_, FDR_PS_, and FDR_D_. The numbers of PSMs for FDR_R_, FDR_PR_, and FDR_D_ were nearly identical, but the numbers of PSMs for FDR_S_ and FDR_PS_ were about 9% (on average) lower than those for FDR_R_, FDR_PR_, and FDR_D_.

### The ratio of unique and redundant peptides in the S. cerevisiae and the human protein database

We compared the ratio of unique (non-redundant) peptides in the target database and various decoy databases to analyze the cause of FDR overestimation for FDR_S_ and FDR_PS_ in the HEK293 datasets. Unique peptides were generated with the following parameters: missed cleavage = 2, min length = 2, max length = 45, and NTT = 2. As shown in Figs. 1a and 1b, in the *S. cerevisiae* protein database, the ratios of unique targets and unique decoy peptides in the reverse database are nearly identical at 49.97:50.03. Shuffle databases have a ratio of 47.19:52.81 on average, the ratios for the pseudo-reverse and de Bruijn databases are 50.05:49.95 and 49.97:50.03 (on average), and the ratio for the pseudo-shuffle database is 48.45:51.55 on average. In the human protein database, the ratio of unique targets and unique decoy peptides for the reverse database is 49.91:50.09, the ratios for the pseudo-reverse and de Bruijn databases are 50.06:49.94 and 49.70:50.30 (on average), whereas shuffle databases have a ratio of 18.02:82.98 on average, and pseudo-shuffle databases show a ratio of 19.19:80.81 on average. (The ratios of another four shuffle databases, four pseudo-shuffle databases, and four de Bruijn database are shown in Supplementary Figure 1).

We found that when the (pseudo) shuffle decoy database is used, the ratios of unique peptides of a target database and a decoy database differ significantly in the human protein database. To find the reason for this, the ratios of redundant peptides in the *S. cerevisiae* and human target databases were compared. A redundant peptide refers to an overlapping peptide from among all peptides in the target database. For example, when protein A is “ATCDEFRGHIPKLNP” and protein B is “YKLMNWRGHIPK,” the tryptic peptide “GHIPK,” which is common to proteins A and B, is termed a redundant peptide. The redundant peptides of the *S. cerevisiae* target database amounted to 7.09% of all peptides, and the redundant peptides in the human target database amounted to 77.38% of all peptides (Fig. 1d).

The ratio of redundant peptides has a considerable influence on the ratio of unique peptides when a decoy database is created using the (pseudo) shuffle method. For example, when there are three overlapping peptides “ACDEFG” in the target database, the (pseudo) reverse method creates three identical peptides “GFEDCA”. Because the overlapping peptides are removed, the unique peptide created when the (pseudo) reverse method is used has only one unique peptide in each of the target and decoy databases. However, given that the (pseudo) shuffle method creates three different peptides, such as “FEGDCA,” “AFEDCG,” and “DCAGEF,” the unique peptides created by the (pseudo) shuffle method consist of one unique peptide in the target database and three unique peptides in the decoy database. Eventually, as the ratio of redundant peptides increases, if the decoy database is created using the (pseudo) shuffle method, an imbalance of unique peptides occurs, as shown in Figs. 1a and 1b. As a result, as shown in Figs. 1b and 1c, FDR_S_ and FDR_PS_ have fewer PSMs compared to FDR_R_, FDR_PR_, and FDR_D_.

### The correction factor is needed when estimating the FDR

We compared the 1% FDR results with the correction factor proposed by Elias and Gygi (Factor 1) [7] and that by Kim et al*.* (Factor 2) [14] and without the correction factor. As shown in Fig. 1a and in Supplementary Figure 1b, in the *S. cerevisiae* protein database, FDR_R_ and FDR_PR_ with the correction factor showed about -0.14 and 0.08% (on average) for the *S. cerevisiae* Elite dataset, and about -0.07 and -0.12% (on average) for the *S. cerevisiae* 2DLC dataset more(more less) PSMs compared to those without the correction factor. In addition, FDR_S_, FDR_PS_ and FDR_D_ with the correction factor showed corresponding increases in the number of PSMs of about 0.89, 0.64 and -0.19% (on average) for the *S. cerevisiae* Elite dataset and about 0.67, 0.43, and -0.07% (on average) for the *S. cerevisiae* 2DLC dataset.

As presented in Figs. 1b and c, for the human protein database, FDR_R_, FDR_PR_, and FDR_D_ with the correction factor showed increases in the number of PSMs of about -0.06, -0.02, and -0.17% (on average) for the HEK293 3-replicate dataset, and by about 0.0%, 0.0% (identical), and -0.23% (on average) for the HEK293 24-fraction dataset. On the other hand, FDR_S_ and FDR_PS_ with the correction factor showed increases in the number of PSMs by about 7.82 and 7.74% (on average) for the HEK293 3-replicate dataset and by about 12.20 and 12.58% (on average) for the HEK293 24-fraction dataset on average. (The results of another four shuffle databases, four pseudo-shuffle databases, and four de Bruijn databases are presented in Supplementary Figure 1) Additionally, as shown in Supplementary Figure 2, FDR_R_, FDR_PR_, FDR_D_ with the correction factor showed increases in the number of PSMs by about -0.12,- 0.08 and -0.14% (on average) for the ten cell line datasets. On the other hand, FDR_S_ and FDR_PS_ with the correction factor showed increases in the number of PSMs by about 12.35 and 12.46% (on average) for ten cell line datasets on average.

These results indicate that FDR_R_, FDR_PR_, and FDR_D_ in both the *S. cerevisiae* and human protein databases showed slight differences regardless of whether or not the FDR was estimated with the correction factor. In the *S. cerevisiae* protein database, there was little difference between FDR_S_ (and FDR_PS_) with the correction factor and FDR_S_ (and FDR_PS_) without the correction factor. However, in the human database, when FDR_S_ (and FDR_PS_) with the correction factor and FDR_S_ (and FDR_PS_) without the correction factor were compared, we found that the number of PSMs for FDR_S_ (and FDR_PS_) with the correction factor increased significantly. In other words, in the human protein database, if the FDR was estimated using the (pseudo) shuffle database without the correction factor, it was overestimated. Accordingly, it is important to estimate the FDR with the correction factor.

### The *S. cerevisiae* and the human six frame translation database

**Fig. 2:**
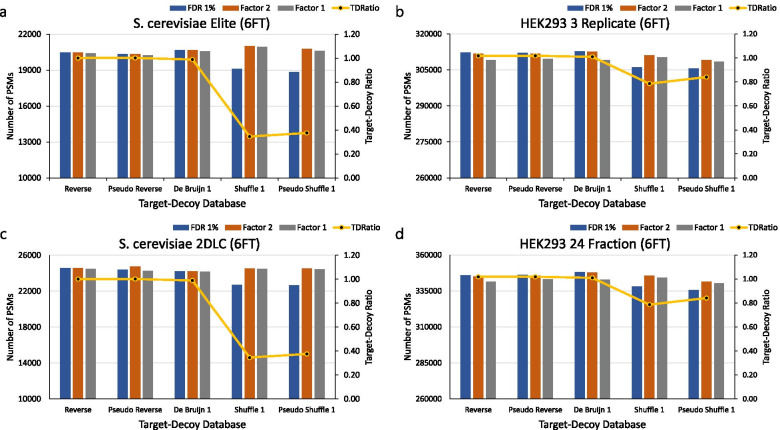
Comparison of the numbers of PSMs of various databases of large size (six-frame translation). The blue bars show the numbers of PSMs for 1% FDR. The orange bars show the numbers of PSMs for 1% FDR using *Factor*2. The gray bars show the numbers of PSMs for 1% FDR using *Factor* 1. The yellow line show the ratio of target and decoy unique peptide ratio. **a** The *S. cerevisiae* six frame translation protein database and *S. cerevisiae* Elite dataset. **b** The human six frame translation protein database and the HEK293 3-Replicate dataset. **a** The *S. cerevisiae* six frame translation protein database and *S. cerevisiae* 2DLC dataset. **b** The human six frame translation protein database and the HEK293 24-Fraction dataset

We used 6FT databases to analyze the degree of FDR overestimation for FDR_S_ and FDR_PS_ in large databases. First, the ratio of unique peptides in the target database and various decoy databases is compared for 6FT databases. Unique peptides were generated with the following parameters: missed cleavage = 2, min length = 2, max length = 45, and NTT = 2. As shown in Fig. 2, in the *S. cerevisiae* 6FT database, the ratio of unique targets and unique decoy peptides in the reverse, pseudo reverse and the de Bruijn databases are nearly identical at 50.03:49.97, 50.04:49.96, and 49.72:50.28, respectively. For *S. cerevisiae*, the shuffle and pseudo shuffle databases have ratios of 25.67:74.33 and 27.30:72.70, respectively. In the human 6FT database, the ratio of unique target and unique decoy peptides in the reverse, pseudo reverse and de bruijn database are nearly identical at 50.45:49.55, 50.45:49.55, and 50.24:49.76, respectively. The shuffle and pseudo shuffle databases have corresponding ratios of 44.02:55.98 and 45.67:54.33.

As shown in Supplementary Figure 3, these results indicate that FDR_R_, FDR_PR_, FDR_D_ in both the *S. cerevisiae* and human protein databases showed slight differences regardless of whether or not the FDR was estimated with the correction factor. However, in the *S. cerevisiae* 6FT database, FDR_S_ and FDR_PS_ with the correction factor showed corresponding increases in the number of PSMs by about 9.81 and 9.90% (on average) for the *S. cerevisiae* Elite dataset, and by about 7.88 and 8.29% (on average) for the *S. cerevisiae* 2DLC dataset. In the human 6FT database, FDR_S_ and FDR_PS_ with the correction factor showed corresponding increases in the number of PSMs by about 1.62 and 1.10% (on average) for the HEK293 3-replicate dataset, and by about 2.22 and 1.73% (on average) for the HEK293 24-fraction dataset.

In addition, we ran a comparison using the separate FDR [15] which is often used in proteogenomics. For the separate FDR, we divided known databases and novel databases. It is easy to divide known and novel databases for the reverse, pseudo reverse, De bruijn decoy databases, but not for the shuffle and pseudo shuffle decoy databases. We note that the known database is the UniProt database and its decoy database and the novel database is the 6FT database apart from the known database. In addition, we calculated the correction factor using these known and novel databases. As shown in Supplementary Figure 3 and Supplementary Table 2, FDR_R_, FDR_PR_ and FDR_D_ of the *S. cerevisiae* and human protein databases show the same number of PSMs, even if the correction factor is used. In the *S. cerevisiae* 6FT database, FDR_S_ and FDR_PS_ with the correction factor showed corresponding increases in the number of PSMs by about 0.93 and 0.31% in known PSMs and by about 35.00 and 8.70% in novel PSMs for the *S. cerevisiae* Elite dataset, also showing corresponding increases in the number of PSMs of about 0.77 and 0.46% in the known PSMs and of about 11.24 and 81.13% in the novel PSMs for the *S. cerevisiae* 2DLC dataset. In the human 6FT database, FDR_S_ and FDR_PS_ with the correction factor showed corresponding increases in the number of PSMs by about 5.82 and 6.00% in the known PSMs and by about 0.03 and 0.00% (identical) for the novel PSMs for the HEK293 3-replicate dataset, and showed increases in the number of PSMs by about 12.32%, 13.07% in known PSMs and by about 2.71%, 0.00% (the same) in novel PSMs for the HEK293 24-fraction dataset. We used a simple method to divide known and novel databases. However, it is likely difficult to divide known and novel databases for the separate FDR. In proteogenomics for the separate FDR, we do not recommend the use of the shuffle and pseudo shuffle decoy databases, because it is difficult to divide known and novel database.

## Conclusion

We used various decoy construction methods in the *S. cerevisiae* and human protein databases to comparison the results of the 1% FDR. When the ratio of redundant peptides in the target database is low (that is, when the ratio of unique peptides in the target and decoy databases is nearly identical), such as in the *S. cerevisiae* protein database, regardless of which decoy construction method is used, the FDR is not overestimated. However, if the ratio of redundant peptides in the target database is high (i.e., the ratio of unique peptides in the target and decoy databases differs significantly), such as in a human protein database, if the (pseudo) shuffle method is used, the FDR is overestimated. Therefore, the FDR must be estimated using the correction factor to avoid FDR overestimation. Additionally, the human and *S. cerevisiae* six frame translation databases, which are large databases, also showed outcomes similar to that of the UniProt human protein database. In contrast, in proteogenomics, we do not recommend the use of shuffle and pseudo shuffle decoy databases for the separate FDR given that, it is difficult to divide known and novel databases, as mentioned above. Additional research is needed to devise a new method capable of dividing known and novel databases.

## Supplementary Information

**Additional file 1: Supplementary Table 1.** The number of MS/MS spectra of the each data set. **Supplementary Table 2.** The number of Known and Novel PSMs. **Supplementary Figure 1.** Comparison of the numbers of PSMs of various databases. The blue bars show the numbers of PSMs for 1% FDR without the correction factor. The orange bars show the numbers of PSMs for 1% FDR using *Factor* 2. The gray bars show the numbers of PSMs for 1% FDR using *Factor* 1. (a) The UniProt *S. cerevisiae* protein database and the *S. cerevisiae* Elite dataset. (b) The UniProt *S. cerevisiae* protein database and the S. cerevisiae 2DLC dataset. (c) The UniProt human protein database and the HEK293 3-replicate dataset. (d) The UniProt human protein database and the HEK293 24-fraction dataset. **Supplementary Figure 2.** Comparison of the numbers of PSMs of various databases. The blue bars show the numbers of PSMs for 1% FDR without the correction factor. The orange bars show the numbers of PSMs for 1% FDR using *Factor* 2. The gray bars show the numbers of PSMs for 1% FDR using *Factor* 1. All databases use the UniProt human protein database. (a) A549 dataset. (b) GAMG dataset. (c) HeLa dataset. (d) HepG2 dataset. (e) JurKat dataset. (f) K562 dataset. (g) LnCap dataset. (h) MCF7 dataset. (i) RKO dataset. (j) U2OS dataset. **Supplementary Figure 3.** Comparison of the numbers of PSMs of various databases. The blue bars show the numbers of PSMs for 1% FDR without the correction factor. The red bars show the numbers of known PSMs for 1% FDR using *Factor* 2. The yellow bars show the numbers of novel PSMs for 1% FDR without the correction factor. The green bars show the numbers of novel PSMs for 1% FDR using *Factor* 2. The black and gray line show the ratio of target and decoy unique peptides ratio of known and novel database, respectively. (a) The *S. cerevisiae* six frame translation protein database and S. cerevisiae Elite dataset. (b) The human six frame translation protein database and the HEK293 3-Replicate dataset. (c) The *S. cerevisiae* six frame translation protein database and S. cerevisiae 2DLC dataset. (d) The human six frame translation protein database and the HEK293 24-Fraction dataset.

## Data Availability

The 11 human cell lines (A549, GAMG, HEK293, HeLa, HepG2, Jurkat, K562, LnCap, MCF7, RKO and U2OS, each 3 replicates) data and HEK293 24 fraction data is publicly available from https://www.ebi.ac.uk/pride/archive/ using PXD002395 and PXD001468, respectively and *Saccharomyces cerevisiae* data is publicly available from https://chorusproject.org/anony-mous/download/experiment/-8823069691100997209. Decoy database generation software is publicly available from https://github.com/othertics/decoygenerator.

## Abbreviations

FDR: False discovery rate
TDS: Target-decoy search strategy
PSM: Peptide-spectrum match
MS/MS: Tandem mass spectrometry
NTT: Number of tryptic termini
HCD: Higher-energy collisional dissociation
*S. cerevisiae*: *Saccharomyces cerevisiae*
