# Supplementary material for: Comparison of false-discovery rates of various decoy databases
Source: Proteome Sci. 2021 Sep 18;19:11. doi: 10.1186/s12953-021-00179-7 (PMC8449453; doi:10.1186/s12953-021-00179-7)
Supplement: Supplementary file 1 — Additional file 1: Supplementary Table 1. The number of MS/MS spectra of the each data set. Supplementary Table 2. The number of Known and Novel PSMs. Supplementary Figure 1. Comparison of the numbers of PSMs of various databases. The blue bars show the numbers of PSMs for 1% FDR without the correction factor. The orange bars show the numbers of PSMs for 1% FDR using Factor 2. The gray bars show the numbers of PSMs for 1% FDR using Factor 1. (a) The UniProt S. cerevisiae protein database and the S. cerevisiae Elite dataset. (b) The UniProt S. cerevisiae protein database and the S. cerevisiae 2DLC dataset. (c) The UniProt human protein database and the HEK293 3-replicate dataset. (d) The UniProt human protein database and the HEK293 24-fraction dataset. Supplementary Figure 2. Comparison of the numbers of PSMs of various databases. The blue bars show the numbers of PSMs for 1% FDR without the correction factor. The orange bars show the numbers of PSMs for 1% FDR using Factor 2. The gray bars show the numbers of PSMs for 1% FDR using Factor 1. All databases use the UniProt human protein database. (a) A549 dataset. (b) GAMG dataset. (c) HeLa dataset. (d) HepG2 dataset. (e) JurKat dataset. (f) K562 dataset. (g) LnCap dataset. (h) MCF7 dataset. (i) RKO dataset. (j) U2OS dataset. Supplementary Figure 3. Comparison of the numbers of PSMs of various databases. The blue bars show the numbers of PSMs for 1% FDR without the correction factor. The red bars show the numbers of known PSMs for 1% FDR using Factor 2. The yellow bars show the numbers of novel PSMs for 1% FDR without the correction factor. The green bars show the numbers of novel PSMs for 1% FDR using Factor 2. The black and gray line show the ratio of target and decoy unique peptides ratio of known and novel database, respectively. (a) The S. cerevisiae six frame translation protein database and S. cerevisiae Elite dataset. (b) The human six frame translation protein database and the HEK293 3-Replicate dataset. (c) [file 12953_2021_179_MOESM1_ESM.docx]

**Comparison of False-Discovery Rate of Various Decoy Databases**

**Supporting Information**

Sangjeong Lee^1^, Heejin Park^1,*^, Hyunwoo Kim^2,*^

^1^ Department of Computer Science, Hanyang University, Seoul 06978, Republic of Korea

^2^ Research Data Sharing Center, Korea Institute of Science and Technology Information, Daejeon 34141, Republic of Korea

Email:

Sangeong Lee: [othertics@hanyang.ac.kr](mailto:othertics@hanyang.ac.kr)

Heejin Park: hjpark@hanyang.ac.kr

Hyunwoo Kim: [pardess@kisti.re.kr](mailto:pardess@kisti.re.kr)

*Corresponding author.

Heejin Park, Email: hjpark@hanyang.ac.kr

Hyunwoo Kim, Email: pardess@kisti.re.kr

**Supplementary Table 1.** The number of MS/MS spectra of the each data set

|  | Number of MS/MS spectra |
| --- | --- |
| A549 | 514,912 |
| GAMG | 625,351 |
| HEK293 | 624,108 |
| HeLa | 543,763 |
| HepG2 | 511,958 |
| Jurkat | 571,069 |
| K562 | 454,612 |
| LnCap | 639,927 |
| MCF7 | 640,530 |
| RKO | 502,079 |
| U2OS | 641,214 |
| HEK293 24 Fraction | 1,121,149 |
| Saccharomyces cerevisiae Elite | 39,444 |
| Saccharomyces cerevisiae 2DLC | 63,031 |

**Supplementary Table 2.** The number of Known and Novel PSMs.

| 6FT | Known | | Novel | |
| --- | --- | --- | --- | --- |
| HEK293 3 Replicate | Without correction factor | With  correction factor | Without correction factor | With  correction factor |
| Reverse | 372647 | 372647 | 399 | 399 |
| Pseudo Reverse | 372536 | 372536 | 449 | 449 |
| De Bruijn | 372710 | 372710 | 445 | 445 |
| Shuffle | 351629 | 372093 | 410 | 423 |
| Pseudo Shuffle | 350817 | 371880 | 146 | 146 |

| 6FT | Known | | Novel | |
| --- | --- | --- | --- | --- |
| HEK293 24 Fraction | Without correction factor | With  correction factor | Without correction factor | With  correction factor |
| Reverse | 464578 | 464578 | 335 | 335 |
| Pseudo Reverse | 464293 | 464293 | 342 | 342 |
| De Bruijn | 464712 | 464712 | 423 | 423 |
| Shuffle | 413747 | 464714 | 406 | 417 |
| Pseudo Shuffle | 410382 | 464017 | 109 | 109 |

| 6FT | Known | | Novel | |
| --- | --- | --- | --- | --- |
| S. cerevisiae Elite | Without correction factor | With  correction factor | Without correction factor | With  correction factor |
| Reverse | 21086 | 21086 | 28 | 28 |
| Pseudo Reverse | 21162 | 21162 | 23 | 23 |
| De Bruijn | 21554 | 21554 | 31 | 31 |
| Shuffle | 21401 | 21600 | 20 | 27 |
| Pseudo Shuffle | 20741 | 20806 | 23 | 25 |

| 6FT | Known | | Novel | |
| --- | --- | --- | --- | --- |
| S. cerevisiae 2DLC | Without correction factor | With  correction factor | Without correction factor | With  correction factor |
| Reverse | 24806 | 24806 | 84 | 84 |
| Pseudo Reverse | 24680 | 24680 | 37 | 37 |
| De Bruijn | 24779 | 24779 | 107 | 107 |
| Shuffle | 24895 | 25087 | 89 | 99 |
| Pseudo Shuffle | 24529 | 24641 | 53 | 96 |


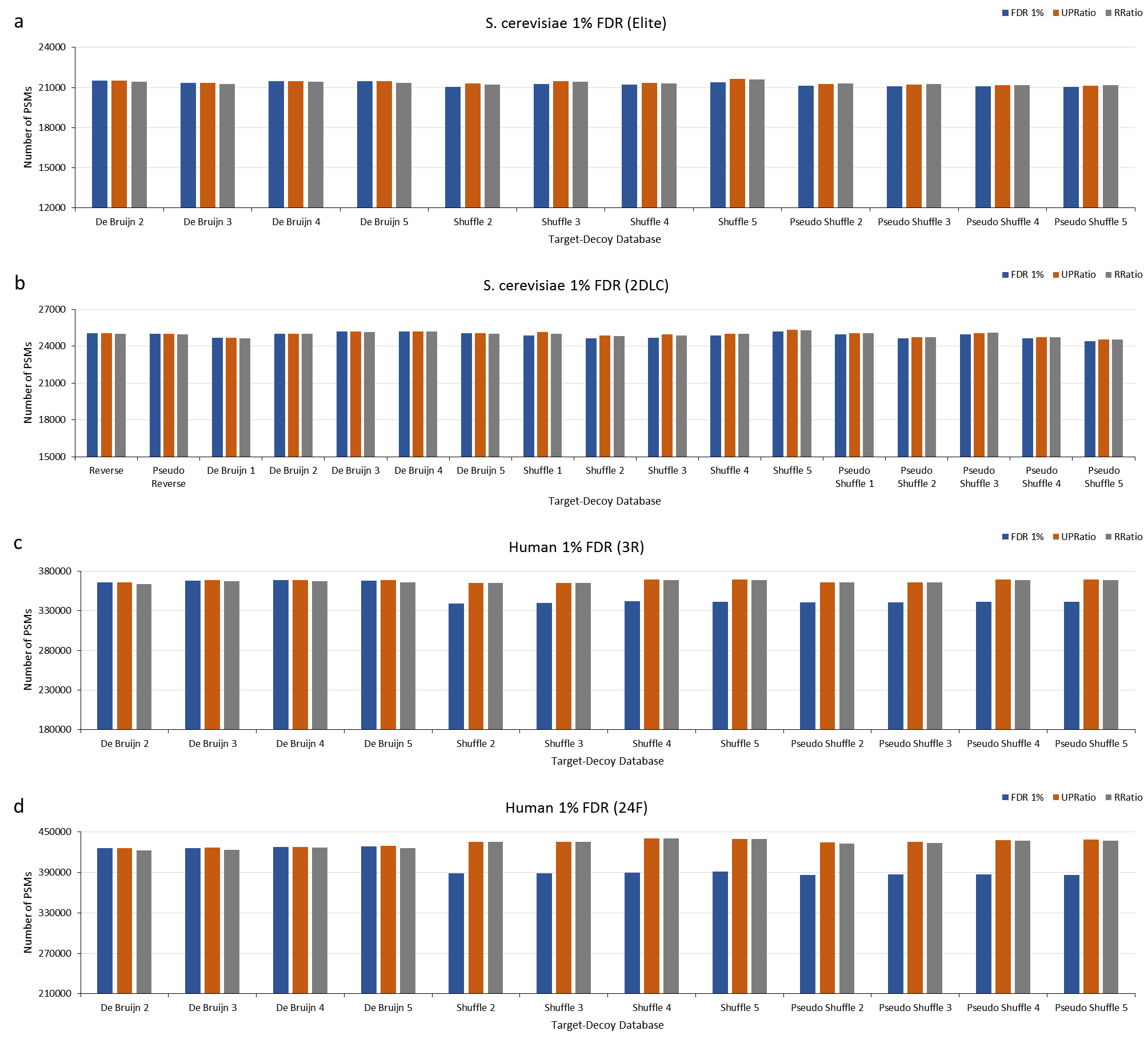


**Supplementary Figure 1.** Comparison of the numbers of PSMs of various databases. The blue bars show the numbers of PSMs for 1% FDR without the correction factor. The orange bars show the numbers of PSMs for 1% FDR using *Factor* 2. The gray bars show the numbers of PSMs for 1% FDR using *Factor* 1. (a) The UniProt *S. cerevisiae* protein database and the *S. cerevisiae* Elite dataset. (b) The UniProt *S. cerevisiae* protein database and the S. cerevisiae 2DLC dataset. (c) The UniProt human protein database and the HEK293 3-replicate dataset. (d) The UniProt human protein database and the HEK293 24-fraction dataset.

**
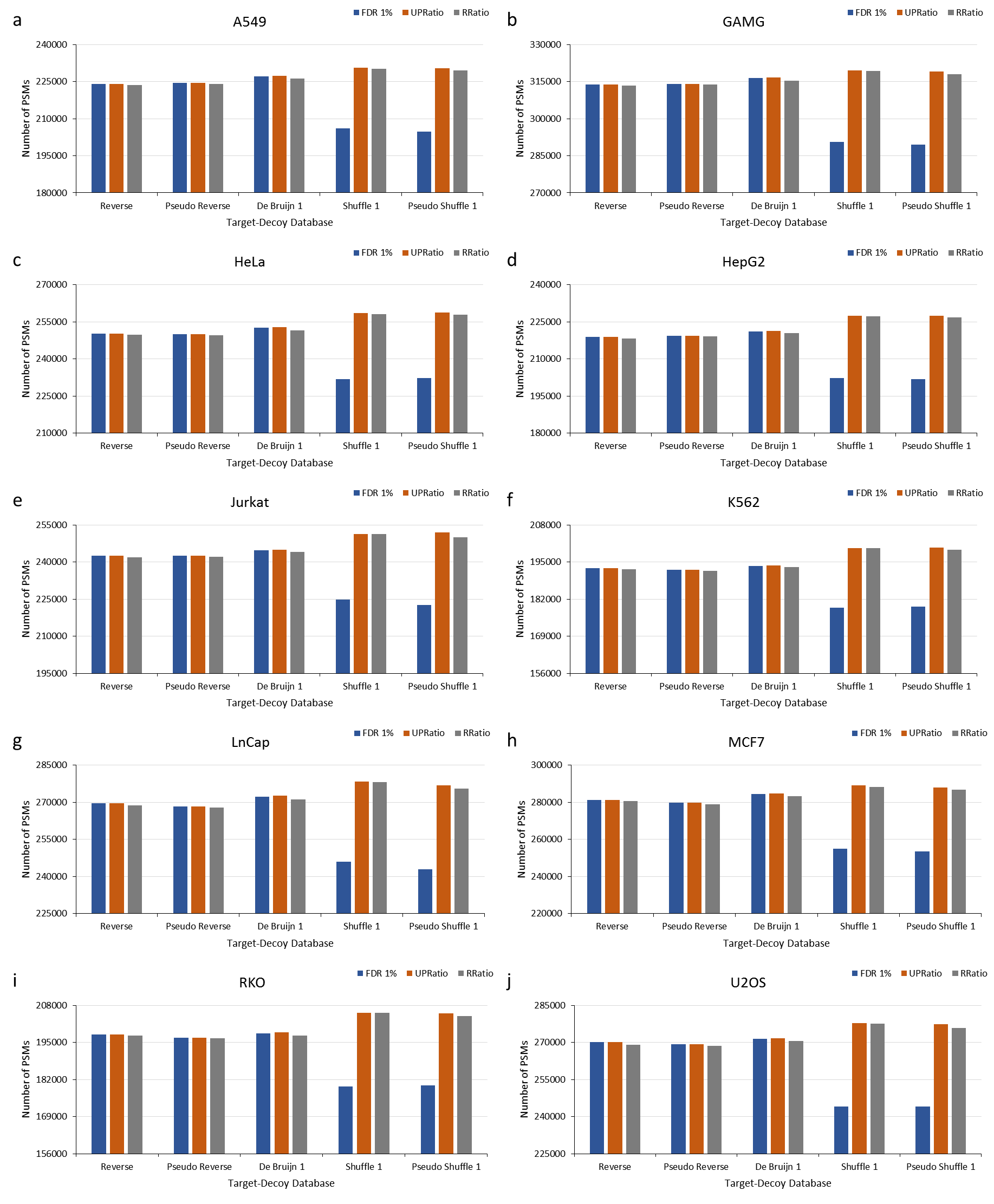
**

**Supplementary Figure 2.** Comparison of the numbers of PSMs of various databases. The blue bars show the numbers of PSMs for 1% FDR without the correction factor. The orange bars show the numbers of PSMs for 1% FDR using *Factor* 2. The gray bars show the numbers of PSMs for 1% FDR using *Factor* 1. All databases use the UniProt human protein database. (a) A549 dataset. (b) GAMG dataset. (c) HeLa dataset. (d) HepG2 dataset. (e) JurKat dataset. (f) K562 dataset. (g) LnCap dataset. (h) MCF7 dataset. (i) RKO dataset. (j) U2OS dataset.


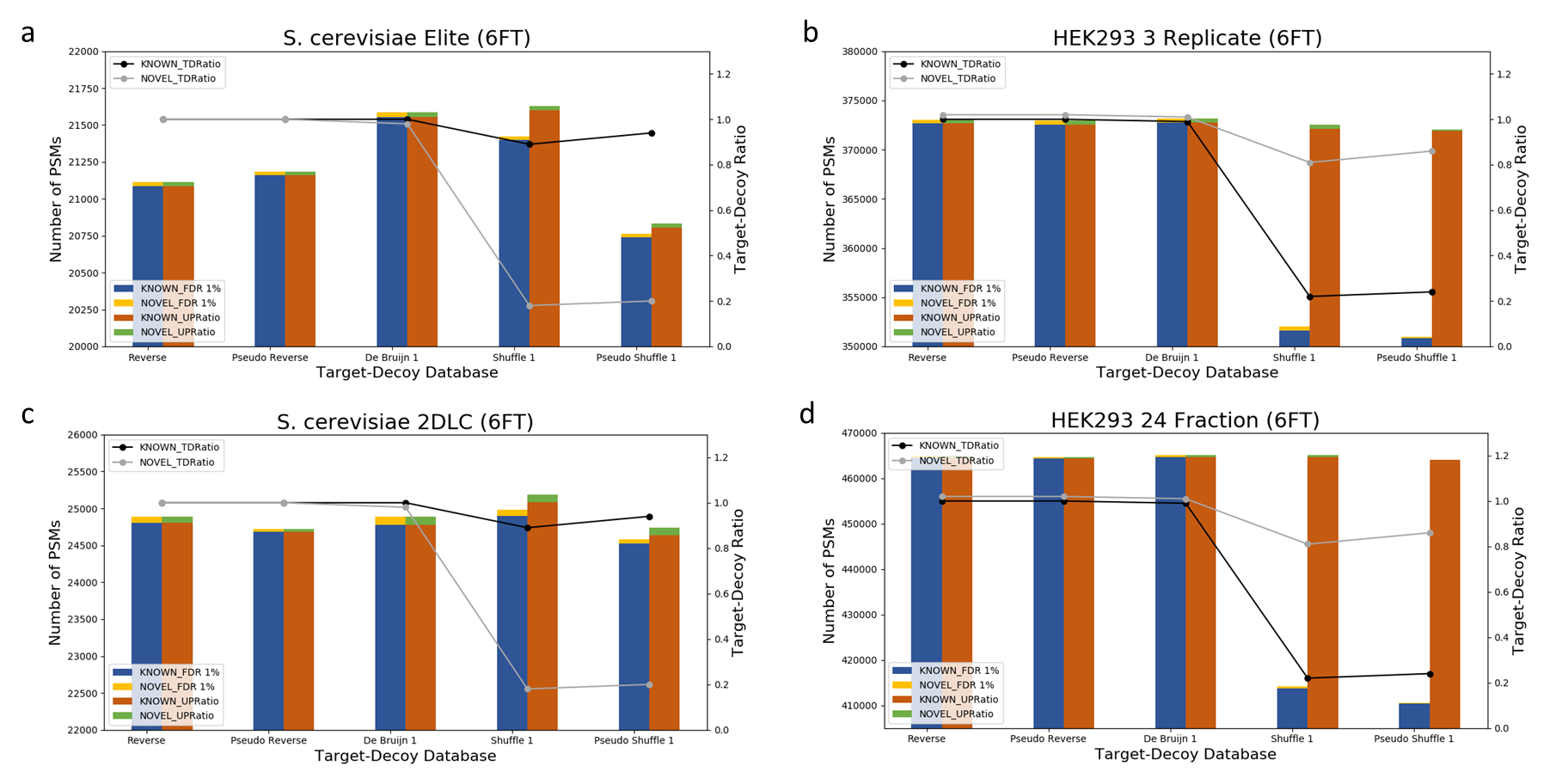


**Supplementary Figure 3.** Comparison of the numbers of PSMs of various databases. The blue bars show the numbers of PSMs for 1% FDR without the correction factor. The red bars show the numbers of known PSMs for 1% FDR using *Factor* 2. The yellow bars show the numbers of novel PSMs for 1% FDR without the correction factor. The green bars show the numbers of novel PSMs for 1% FDR using *Factor* 2. The black and gray line show the ratio of target and decoy unique peptides ratio of known and novel database, respectively. (a) The *S. cerevisiae* six frame translation protein database and S. cerevisiae Elite dataset. (b) The human six frame translation protein database and the HEK293 3-Replicate dataset. (c) The *S. cerevisiae* six frame translation protein database and S. cerevisiae 2DLC dataset. (d) The human six frame translation protein database and the HEK293 24-Fraction dataset.
